# The Impact of a Laparoscopic Surgery Training Course in a Developing Country

**DOI:** 10.1007/s00268-020-05606-y

**Published:** 2020-06-24

**Authors:** Esther Westwood, Balaram Malla, Jeremy Ward, Roshan Lal, Kamal Aryal

**Affiliations:** 1 James Paget University Hospitals NHS Trust, Lowestoft Road, Gorleston-on-Sea, Great Yarmouth, NR31 6LA UK; 2 Dhulikhel Hospital, Dhulikhel, 45200 Nepal; 3 Lancashire Teaching Hospitals NHS Foundation Trust, Sharoe Green Lane, Fulwood, Preston, PR2 9HT UK; 4 East Anglia University, Norwich Research Park, Norwich, NR4 7TJ UK

## Abstract

**Introduction:**

Surgeons training junior colleagues to perform laparoscopic surgery find the ‘apprenticeship’ model of surgical training inadequate. Therefore, the use of training courses involving simulation have become an important way to teach laparoscopic surgery. An annual laparoscopic surgery course began in Nepal in 2013. It is difficult to assess the impact of the course on trainees and demonstrate a subsequent improvement in patient outcomes, but one way is to ask delegates for reflections on their experience of the course and their perception of how it has impacted patients.

**Methods:**

The course involved simulation and patient-based training. A questionnaire to collect quantitative data and qualitative comments was sent to all 80 previous delegates (at least 9 months after the course) in September 2018.

**Results:**

Twenty-eight delegates responded. The majority demonstrated career progression since completing the course (independent practitioners increased from 7 to 50%) and progression in their practice (18% had performed >20 laparoscopic procedures at the time of the course, vs 70% at the time of the questionnaire). All delegates felt that laparoscopic training is useful in the Nepalese context. Delegates felt the course was useful in developing skills, and improving confidence and safety. Suggestions for improvement included lengthening the course and increasing the amount of practical exposure.

**Conclusion:**

There was a positive outcome of the course to Kirkpatrick level 2. There is a need to expand the course’s scope to an advanced level, increase its length and start courses in other centres, to ensure the most possible benefit to patients.

## Introduction

### Training in laparoscopic surgery

Historically, surgical training has been based around an expert training an apprentice in clinical and surgical skills [1]. However, this approach is no longer considered robust enough to develop the surgeons of tomorrow, who are being trained in increasingly technologically advanced methods. It has been shown to be time-consuming, costly and does not give consistent results. Furthermore, this approach exposes patients to risk, as it necessitates that trainees perform skills that they have never done before on real patients [2].

A major change in the practice of surgery in the last 30 years has been the introduction and widespread use of laparoscopic surgery: laparoscopy is now considered the gold standard of care for many surgical procedures [3].

The difficulty of maintaining the apprenticeship approach in combination with laparoscopic surgical training is that laparoscopic surgery has a steeper learning curve than open surgery, and each individual procedure has a different learning curve [1, 4]. Therefore, the training that a trainee receives in clinical practice will largely depend on the patients that come through the door of the hospital [5].

Happily, laparoscopic surgery lends itself to other training methods, in the form of a variety of simulation techniques. These include ‘dry’ laboratory techniques, such as box models and virtual reality simulators, and ‘wet’ laboratory techniques, such as animal or cadaver models [3]. Simulation training has been shown to result in transfer of skills to the operative environment with no risk to patients [4]. It softens the steep learning curve of laparoscopic skills and allows trainees to develop confidence outside the operating theatre [6].

### Surgical training in the developing world

There is a great need for effective surgical training in the developing world, as it is estimated that up to half the world’s population does not have access to basic surgical care. This phenomenon is exacerbated by brain drain from developing countries to developed countries as surgical trainees seek opportunities for training abroad. Attempts to remedy the lack of access to safe surgery (including short-term aid by surgeons from developed countries and trainees from developed countries spending part of their training in developing countries) have not successfully addressed the problem. A more sustainable approach has been found in the development of local training programmes, which produce competent local surgeons who are more inclined to stay in the developing country [7].

### The Nepalese context

One such developing country is Nepal, a land-locked country in south Asia. Nepal faces many challenges in delivering surgical care to its population, due to the large numbers of the population living in rural areas, political unrest causing stagnation of the development of a national health service and poverty resulting in a lack of resources. However, in spite of this, opportunities for undergraduate and postgraduate medical training have been increasing in recent years, with a subsequent increase in the number of doctors wishing to undertake surgical training. Sadly, there is a lack of consistency between postgraduate training programmes, and therefore, a need for structured, quality assured training in surgery [8].The difficulties in delivering training in laparoscopic surgery in particular are exacerbated by the high cost of instruments and equipment, and the need for a change in the professional culture amongst Nepalese surgeons to accept a relatively new surgical technique.

There can be no doubt of the need for laparoscopic surgery in Nepal, as its benefits are clear: smaller incisions lead to reduced pain and a shorter inpatient stay as compared with open surgery. This is of particular relevance to the Nepalese population, many of whom have to travel some distance from remote villages for surgery, and rely on a rapid return to physical fitness in order to maintain a living as manual labourers.

With this in mind, a laparoscopic surgery training course was set up in Nepal in 2013. The course has been run annually and delivers a combination of didactic teaching, dry laboratory simulations and supervised live operating at Dhulikhel Hospital near Kathmandu. The teaching is led by experienced surgeons from the United Kingdom and Nepal. The delegates are recently qualified surgeons from Nepalese Universities who work in variety of settings: some in large teaching hospitals in Kathmandu and others in smaller district hospitals in more remote areas. The delegates should have been involved in 5–10 laparoscopic procedures prior to participating in the course. The course is accredited and quality controlled by the Royal College of Surgeons of Edinburgh (RCSEd) [9]. The learning outcomes of the course are shown in Box 1.

**Box 1:** Learning outcomes of the laparoscopic surgery training course

Discuss case selection, principles, techniques, benefits and complications of laparoscopic appendicectomy, laparoscopic cholecystectomy and laparoscopic hernia repair and laparoscopic right hemicolectomy
Suture laparoscopically in a simulator
Tie an intracorporeal knot in a simulator

It is difficult to accurately assess the impact of the course, both on the delegates themselves and on the patients that they have subsequently cared for, but this can be attempted by asking delegates to reflect on the course and their perceptions of how it has impacted patient outcomes.

## Methods

### Laparoscopic surgery training course structure

Prior to the laparoscopic surgery training course, delegates are emailed with pre-course learning material detailing physiological changes during laparoscopic surgery, the use of laparoscopic equipment and specifics regarding certain laparoscopic procedures (cholecystectomy, appendicectomy, hernias and colorectal procedures). The face-to-face element of the course is run over 3 days and is delivered by 5 faculty members. Day 1 of the face-to-face course involves 8 interactive lectures around the principles and practice of laparoscopic surgery (including video demonstrations) and a session to introduce laparoscopic simulation. On day 2, the delegates are split into 2 groups of 8 delegates. Group A performs tasks on the laparoscopic simulators in 2 × 3 h sessions, which allow delegates to practice simple tasks (grommet tasks, cutting a circle, needle pass), before progressing to more complex tasks including suturing. Group B practices live operating of common laparoscopic procedures (cholecystectomy, inguinal hernia repair, incisional hernia repair, appendicectomy, right hemicolectomy) under supervision (x at least 1 case per delegate). Delegates are closely monitored and have the opportunity to ask questions. On day 3, delegates take a 40 question multiple choice questionnaire and have the opportunity to receive feedback. Following this, group A practices live operating, while group B performs tasks on the laparoscopic simulators in 2 × 3 h sessions. Following the course, delegates are given videos of standard operations on above procedures performed by the faculty to review in their own time with a document containing the steps of the operation.

### Questionnaire

The delegates’ perception of the course and its impact was assessed by completion of a questionnaire (“Appendix”; N.B. this was in addition to the formal course evaluation required for accreditation by the RCSEd). The questionnaire was sent to all 80 delegates (2013–2018) by email in September 2018 (median time from completion of course to completion of questionnaire 40 months, range 10–70 months), and involved collection of demographic data (stage of training at the time of participation in the course and at the time of completing the questionnaire), quantitative data on procedure numbers performed by each surgeon and scoring on perceived usefulness of the course, and qualitative data in the form of comments on the course and suggestions for improvement. Thematic analysis was carried out on the qualitative comments to assess common themes [10].

## Results

Twenty-eight delegates responded to the questionnaire. The majority had progressed in their career. 15 (55%) were residents, 10 (37%) were registrars (defined as having passed postgraduate examinations but working under supervision) and 2 (7%) were independent practitioners at the time of participation in the course, while 3 (11%) were residents, 11 (39%) were registrars and 14 (50%) were independent practitioners at the time of answering the questionnaire.

Delegates had shown good progression in their level of exposure to laparoscopic surgery (as demonstrated in Table 1) with an increase in procedures performed before and after the course.

**Table 1:** Number of laparoscopic procedures performed by delegates at the time of the courses vs at the time of the study

Number of procedures	At time of course	At time of study
No exposure	2 (7%)	0
Assisted only	9 (32%)	1 (4%)
<5	6 (21%)	2 (7%)
5–10	4 (14%)	2 (7%)
10–20	2 (7%)	3 (11%)
>20	5 (18%)	20 (71%)

**Fig. 1:**
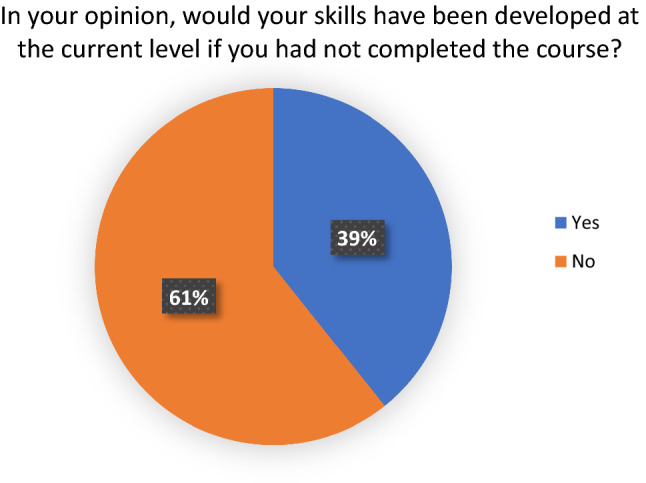
Answers to the question, ‘In your opinion, would your skills have been developed at their current level if you had not completed the course?

**Fig. 2:**
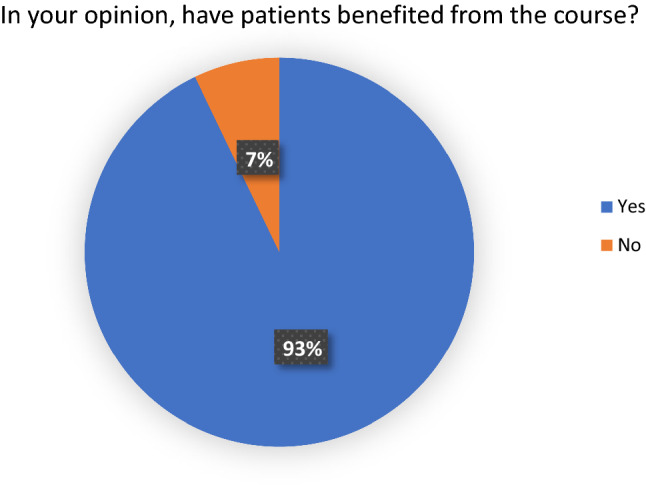
Answers to the question, ‘In your opinion, have the patients benefited from this course?’

**Fig. 3:**
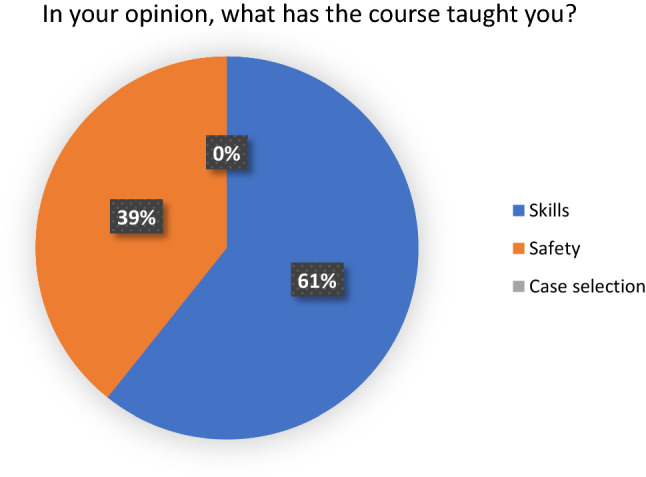
Answers to the question, ‘In your opinion, what has the course taught you?’

**Fig. 4:**
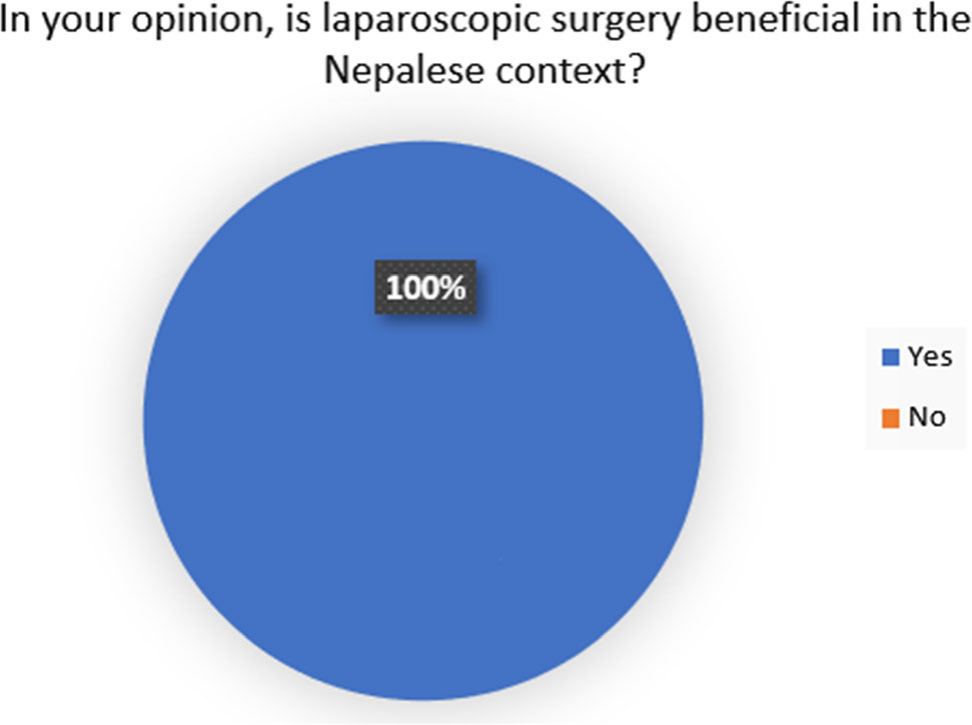
Answers the question, ‘In your opinion, is laparoscopic surgery beneficial in the Nepalese context?’

All delegates felt that laparoscopic surgery training is beneficial in the Nepalese context. 27 delegates (96%) felt that the course had made a positive impact on their practice. 26 delegates (92%) felt that patients benefitted from their attendance on the course. 17 delegates (61%) believed that their skills would not have developed to their current level had they not attended the course (Figs. 1, 2, 3 and 4).

Twenty-six delegates provided an answer to the qualitative question. The feedback showed that delegates found the course useful (/beneficial; *n* = 8) and those that specified in what way the course was useful wrote about building confidence (*n* = 5), improving skill (*n* = 6) and improving safety (*n* = 5). Of the delegates that suggested improvements, the majority wanted the course to be longer (*n* = 6) and more ‘hands on’ (*n* = 4). 1 delegate suggested time in a wet laboratory as an improvement. 3 delegates suggested building on the delivery of the course: 1 suggested expanding the material to cover advanced cases, 1 suggested expanding to other centres and 1 felt that trainees would benefit from the creation of fellowships in laparoscopic surgery.

## Discussion

### Results summary

While far from perfect, post-course questionnaires can be useful in assessing the outcome and impact of medical education courses. The Kirkpatrick Model [11, 12], a method of evaluating the efficacy of training programmes, gives 4 levels to classify the impact of the training: 1—reaction (participant perception); 2—learning (demonstrable change in attitude, knowledge gained, or skills improved); 3—behaviour (change in practice as a result of application of the learning); 4—results (improved outcomes as a result of the learning) [13]. Levels 1 and 2 can be partially assessed by simply asking participants to honestly evaluate the course and their subsequent practice in light of the training received, as was done here. Levels 3 and 4 are more difficult to assess and require more in depth analysis.

From the questionnaire, it is clear that the majority of delegates felt that they had benefited from the course in 3 main areas: building confidence, improving skills and increasing patient safety. Most delegates also felt that patients directly benefited from their attendance on the course—this satisfies Kirkpatrick level 1. The demonstration of the delegates progression in their careers such that the majority have now performed over 20 laparoscopic procedures meets Kirkpatrick level 2.

While assessment of delegates’ perceptions can only go so far, it is likely that feelings on the usefulness of the course do have some grounding in reality, as it is known that structured training programmes do result in safer practice [14]. This has been theorised to stem from improved confidence after practicing in a risk-free environment, as well as significant skill transfer from the laboratory environment to the operating theatre [6].

The other main outcome of the questionnaire was that the delegates have demonstrated a desire for further training (and provided suggestions on how this can be delivered). The most conclusively answered question was ‘In your opinion, is laparoscopic surgery training beneficial in Nepalese context?’ All delegates answered in the affirmative. This shows a clear feeling of a need for further training, and careful thought as to how it could be delivered.

### Limitations

A relatively small number of delegates responded the questionnaire (28 delegates from a total of 80). The delegates that responded may also have been subject to selection bias: delegates who found the course most beneficial would be more likely to respond and share their views.

A further limitation lies in the questionnaire as a means of gathering feedback, as it is low fidelity and cannot adequately satisfy anything beyond Kirkpatrick level 2.

### Next steps

Some delegates suggested including wet laboratory training into the course, and this option is currently being explored. It will require an expansion to the hospital, which is currently being considered. This would require collaboration and willingness from the Nepalese government, universities and surgical departments in order to realise nationwide improvements in surgical care, and ultimately, improved outcomes for Nepalese patients.

## Conclusion

The course was well received by the majority of delegates, who have shown significant progression in their practice and career since the course. Nearly all the delegates felt that patients benefited from their attendance on the course, with a few delegates specifically mentioning an increase in skill and safety when performing laparoscopic surgery following the course, despite its short length. The suggestions for improvement demonstrate a hunger amongst the Nepalese trainees for more high-quality laparoscopic surgery training, which could be delivered through an extended version of this course, or perhaps by training local clinicians to facilitate regular laparoscopic skills and simulation training, with the addition of wet laboratory training.

## Conflict of interest

The authors declare that they have no conflict of interest.

## Appendix 1

Survey Questions.

1. What is your current level of practice?
  - Resident (including medical officer grade)
  - Registrar grade (passed postgraduate exams but work under supervision)
  - Independently practicing surgeon at consultant level
2. What was your level of practice when you did the course?
  - Resident (including medical officer grade)
  - Registrar grade (passed postgraduate exams but work under supervision)
  - Independently practicing surgeon at consultant level
3. How many laparoscopic surgery cases you had performed before you participated in the course? (performed means either performed independently or under supervision)
  - No exposure
  - Assisted only
  - Performed less than 5
  - Performed 5–10
  - Performed 10–20
  - Performed more than 20
4. How many laparoscopic surgery cases have you performed since attending the curse? (performed means either performed independently or under supervision)
  - No exposure
  - Assisted only
  - Performed less than 5
  - Performed 5–10
  - Performed 10–20
  - Performed more than 20
5. In your opinion, has the course made a positive impact on your practice?
  - Yes
  - No
6. In your opinion, would your skills been developed at the current level if you had not completed the course?
  - Yes
  - No
7. In your opinion, have the patients benefited from this course?
  - Yes
  - No
8. In your opinion what has the course taught you?
  - Skills
  - Safety
  - Case selection
9. In your opinion, is laparoscopic surgery training beneficial in Nepalese context?
  - Yes
  - No
10. Please give your honest opinion about usefulness and impact of this course
